# On Pillai’s Problem involving Lucas sequences of the second kind

**DOI:** 10.1007/s40993-024-00534-5

**Published:** 2024-05-13

**Authors:** Sebastian Heintze, Volker Ziegler

**Affiliations:** 1 Institute of Analysis and Number Theory, Graz University of Technology, Steyrergasse 30/II, A-8010 Graz, Austria; 2 University of Salzburg, Hellbrunnerstrasse 34/I, A-5020 Salzburg, Austria

**Keywords:** Diophantine equations, Pillai’s Problem, Lucas sequences, 11D61, 11B37, 11J86

## Abstract

In this paper, we consider the Diophantine equation $V_n - b^m = c$ for given integers *b*, *c* with $b \ge 2$, whereas $V_n$ varies among Lucas-Lehmer sequences of the second kind. We prove under some technical conditions that if the considered equation has at least three solutions (*n*, *m*) , then there is an upper bound on the size of the solutions as well as on the size of the coefficients in the characteristic polynomial of $V_n$.

## Introduction

In recent times, many authors considered Pillai-type problems involving linear recurrence sequences. For an overview we refer to [5]. Let us note that these problems are inspired by a result due to S. S. Pillai [9, 10] who proved that for given, coprime integers *a* and *b*, there exists a constant $c_0(a,b)$, depending on *a* and *b*, such that for any $c>c_0(a,b)$ the equation

1 $$\begin{aligned} a^n-b^m=c \end{aligned}$$

has at most one solution $(n,m)\in \mathbb {Z}_{>0}^2$.

Replacing the powers $a^n$ and $b^m$ by other linear recurrence sequences seems to be a challenging task which was supposedly picked up first by Ddamulira, Luca and Rakotomalala [4], where it was shown that

$$\begin{aligned} F_n-2^m=c \end{aligned}$$

has at most one solution $(n,m)\in \mathbb {Z}_{>0}^2$ provided that

$$\begin{aligned} c\notin \{0,1,-1,-3,5,-11, -30, 85\}. \end{aligned}$$

More generally, Chim, Pink and Ziegler [3] proved that for two fixed linear recurrence sequences $(U_n)_{n\in \mathbb {N}}$, $(V_n)_{n\in \mathbb {N}}$ (with some restrictions) the equation

$$\begin{aligned} U_n - V_m = c \end{aligned}$$

has at most one solution $(n,m)\in \mathbb {Z}_{>0}^2$ for all $c\in \mathbb {Z}$, except if *c* is in a finite and effectively computable set $\mathcal {C} \subset \mathbb {Z}$ that depends on $(U_n)_{n\in \mathbb {N}}$ and $(V_n)_{n\in \mathbb {N}}$.

In more recent years, several attempts were made to obtain uniform results, i.e. to allow to vary the recurrence sequences $(U_n)_{n\in \mathbb {N}}$ and $(V_n)_{n\in \mathbb {N}}$ in the result of Chim, Pink and Ziegler [3]. In particular, Batte, Ddamulira, Kasozi and Luca [1] showed that for all pairs $(p,c)\in \mathbb {P} \times \mathbb {Z}$ with *p* a prime, the Diophantine equation

$$\begin{aligned} F_n-p^m=c \end{aligned}$$

has at most four solutions $(n,m)\in \mathbb {N}^2$ with $n\ge 2$. This result was generalized by Heintze, Tichy, Vukusic and Ziegler [5]. They proved that for a given linear recurrence sequence $(U_n)_{n\in \mathbb {N}}$ (with irrational simple dominant root $\alpha > 1$ having a positive Binet coefficient *a*, such that $\alpha$ and *a* are multiplicatively independent and such that the equation $\alpha ^z-1=a^x \alpha ^y$ has no solutions with $z \in \mathbb {N}$, $x,y \in \mathbb {Q}$ and $-1<x<0$), there exist an effectively computable bound $B\ge 2$ such that for an integer $b > B$ the Diophantine equation

2 $$\begin{aligned} U_n-b^m=c \end{aligned}$$

has at most two solutions $(n,m)\in \mathbb {N}^2$ with $n\ge N_0$. Here $N_0$ is an effectively computable constant depending only on $(U_n)_{n\in \mathbb {N}}$.

In this paper, we want to fix *b* in (2) and let $(U_n)_{n\in \mathbb {N}}$ vary over a given family of recurrence sequences. In particular, we consider the case where $(U_n)_{n\in \mathbb {N}}$ varies over the family of Lucas-Lehmer sequences of the second kind.

## Notations and statement of the main results

In this paper, we consider Lucas-Lehmer sequences of the second kind, that is we consider binary recurrence sequences of the form

$$\begin{aligned} V_n(A,B)=V_n=\alpha ^n+\beta ^n, \end{aligned}$$

where $\alpha$ and $\beta$ are the roots of the quadratic polynomial

$$\begin{aligned} X^2-AX+B \end{aligned}$$

with $A^2\ne 4B$ and $\gcd (A,B)=1$. In the following, we will assume that $V_n$ is non-degenerate, i.e. that $\alpha /\beta$ is not a root of unity. More precisely, we assume $A^2-4B>0$ and $A\ne 0$, since this implies that $\alpha$ and $\beta$ are distinct real numbers with $|\alpha |\ne |\beta |$. We will also assume that $A>0$, which results in $V_n>0$ for all $n\in \mathbb {N}$. Then we consider the Diophantine equation

3 $$\begin{aligned} V_n-b^m=c, \end{aligned}$$

where $b,c\in \mathbb {Z}$ with $b>1$ are fixed.

### Theorem 1

Let $0<\epsilon <1$ be a fixed real number and *A*, *B* coprime integers satisfying $A^2-4B>0$. Assume that $|B|<A^{2-\epsilon }$ as well as that the polynomial $X^2-AX+B$ is irreducible and $A \ge 32^{1/\epsilon }$. Furthermore, assume that $b\ge 2$ if $c\ge 0$ and $b\ge 3$ if $c<0$. Assume that Eq. (3) has three solutions $(n_1,m_1),(n_2,m_2),(n_3,m_3)\in \mathbb {N}^2$ with $n_1>n_2>n_3\ge N_0(\epsilon )$ for the bound $N_0(\epsilon )=\frac{3}{2\epsilon }$. Then there exists an effectively computable constant $C_1=C_1(\epsilon ,b)$, depending only on $\epsilon$ and *b*, such that $n_1<C_1$. In particular, we can choose

$$\begin{aligned} C_1= 4.83\cdot 10^{32}\frac{(\log b)^4}{\epsilon ^2} \left( \log \left( 5.56\cdot 10^{36} \frac{(\log b)^4}{\epsilon ^2}\right) \right) ^4. \end{aligned}$$

Let us note that the bound $N_0=N_0(\epsilon )$ in Theorem 1 ensures that $V_n$ is strictly increasing for $n\ge N_0$ (see Lemma 9 below). Let us also mention that it is essential to exclude the case that $V_{n_2}=V_{n_3}$.

Although we can bound $n_1$ in terms of *b* and $\epsilon$, our method does not provide upper bounds for *A* and |*B*|. However, in the case that we are more restrictive in the possible choice of *B*, we can prove also upper bounds for *A* and |*B*|.

### Theorem 2

Let $\kappa \ge 1$ be a fixed real number and *A*, *B* coprime integers satisfying $A^2-4B>0$. Assume that $|B|<\kappa A$ as well as that the polynomial $X^2-AX+B$ is irreducible. Furthermore, assume that $b\ge 2$ if $c\ge 0$ and $b\ge 3$ if $c<0$. Assume that Eq. (3) has three solutions $(n_1,m_1),(n_2,m_2),(n_3,m_3)\in \mathbb {N}^2$ with $n_1>n_2>n_3\ge 1$. Then there exist effectively computable constants $C_2=C_2(\kappa ,b)$, depending only on $\kappa$ and *b*, and $C_3=C_3(b)$, depending only on *b*, such that $\log A<\max \{C_2,C_3\}$. We can choose

$$\begin{aligned} C_2=4.35 \cdot 10^{10}\log (4\kappa ) \log b\log \left( 5.98 \cdot 10^{11} \log b\right) \end{aligned}$$

and

$$\begin{aligned} C_3= 9.41\cdot 10^{9} \left( \log \left( 1.4\cdot 10^{36}(\log b)^4\left( \log \left( 2.23\cdot 10^{37}(\log b)^4 \right) \right) ^4\right) \log b \right) ^2. \end{aligned}$$

A straight forward application of our bounds yields:

### Corollary 3

Assume that $|B|<A$. If the Diophantine equation

4 $$\begin{aligned} V_n-2^m=c \end{aligned}$$

with $c\ge 0$ has three solutions $(n_1,m_1),(n_2,m_2),(n_3,m_3)\in \mathbb {N}^2$ with $n_1>n_2>n_3\ge 1$, then either

- $$ A<1024 $$ or
- $$n_1<2.3\cdot 10^{40}$$ and $$\log A<4.48\cdot 10^{13}$$.

Another consequence of Theorem 2 together with the results of Chim, Pink and Ziegler [3] is that there exist only finitely many Diophantine equations of the form of (3) that admit more than two solutions, provided that $|B|<\kappa A$. The following corollary gives a precise statement.

### Corollary 4

Let $\kappa \ge 1$ be a fixed real number and $b\ge 2$ a fixed integer. Then there exist at most finitely many, effectively computable 9-tuples $(A,B,c,n_1,m_1,n_2,m_2$, $n_3,m_3)\in \mathbb {Z}^9$ such that

- *A* and *B* are coprime;
- $$A \ge \max \{\kappa ^2,1024\}$$, $$|B|< \kappa A$$ and $$A^2-4B>0$$;
- $$X^2-AX+B$$ is irreducible;
- if $$b=2$$, then $$c\ge 0$$;
- $$n_1>n_2>n_3\ge 1$$ and $$m_1,m_2,m_3\ge 1$$;
- $$V_{n_i}(A,B)-b^{m_i}=c$$ for $$i=1,2,3$$.

### Proof

For any fixed value of *c*, we apply Theorem 2 and get an upper bound for *A*, independent of *c*. So there remain only finitely many, effectively computable possibilities for *A* and *B*. Thus we have finitely many possibilities for the recurrence $V_n$. Chim, Pink and Ziegler [3] proved that, for two in absolute values strictly increasing linear recurrences $(X_n)_{n\ge N_0}$ and $(Y_m)_{m\ge M_0}$ with multiplicatively independent dominant roots, there exists only finitely many, effectively computable integers *c* such that $X_n - Y_m = c$ has more than one solution. First, we have to ensure that $\alpha$ and *b* are multiplicatively independent. However, $\alpha$ and *b* are multiplicatively dependent if and only if there exist integers *x*, *y* not both zero such that $\alpha ^x=b^y$. But $\alpha ^x$ cannot be a rational number, unless $x=0$, since by our earlier assumptions $A>0$ and $A^2-4B > 0$ is not a perfect square. Second, we note that $V_n$ is strictly increasing for $n \ge 1$ by Lemma 10 since $A \ge \max \{\kappa ^2,1024\}$ implies $A \ge 4\kappa +4$. Therefore we can apply their result in our situation and obtain that there are only finitely many, effectively computable values for *c*. Finally, we use Theorem 1 with $\epsilon =\frac{1}{2}$ to bound the $n_i$. Clearly, the $m_i$ can now be calculated. $\square$

Let us give a quick outline for the rest of the paper. In the next section we establish several lemmas concerning properties of Lucas sequences $V_n$ under the restrictions that $|B|<A^{2-\epsilon }$ and $|B|<\kappa A$, respectively, that we will frequently use throughout the paper. The main tool for the proofs of the main theorems are lower bounds for linear forms in logarithms of algebraic numbers. In Sect. 6 we establish bounds for $n_1$, which still depend on $\log \alpha$, following the usual approach (cf. [3]). In Sect. 7, under the assumption that three solution exist, we obtain a system of inequalities involving linear forms in logarithms which contain $\log \alpha$. Combining these inequalities we obtain a linear form in logarithms which does no longer contain $\log \alpha$. Thus we obtain that Theorems 1 and 2 hold or one of the following two cases occurs:

- $$n_1m_2-n_2m_1=0$$;
- $$m_2-m_3\ll \log n_1$$.

That each of these cases implies Theorems 1 and 2 is shown in the subsequent Sects. 8 and 9.

## Auxiliary results on Lucas sequences

Let $\alpha$ and $\beta$ be the roots of $X^2-AX+B$. By our assumptions, $\alpha$ and $\beta$ are distinct real numbers $\notin \mathbb {Q}$. Throughout the rest of the paper, we will assume that $\alpha$ is the larger one, i.e. $|\alpha |>|\beta |$, which we can do since, by our assumptions, we have $A > 0$. Therefore we obtain

$$\begin{aligned} \alpha =\frac{A+\sqrt{A^2-4B}}{2} \qquad \text { and } \qquad \beta =\frac{A-\sqrt{A^2-4B}}{2}. \end{aligned}$$

First, note that $\alpha >1$ can be bounded in terms of *A*:

### Lemma 5

Assuming that $|B|<A^2$, we have $\frac{A}{2}<\alpha <2A$.

At some point in our proofs of Theorems 1 and 2, we will use that $\beta$ cannot be to close to 1. In particular, the following lemma will be needed.

### Lemma 6

Let $A, B, \alpha , \beta$ be as above, in particular $X^2-AX+B$ irreducible with real roots. Assume that $A\ge 4$. Then we have

$$\begin{aligned} \left| 1-|\beta |\right| \ge \frac{2}{2A+5}. \end{aligned}$$

### Proof

First, let us note that the function $f(x)=\frac{A-\sqrt{A^2-4x}}{2}$ is strictly increasing for all $x<A^2/4$. Moreover, we have $f(A-1)=1$ and $f(-A-1)=-1$. By our assumption that $X^2-AX+B$ is irreducible, a choice for *B* such that $|\beta |=1$ is not admissible. Therefore we have

$$\begin{aligned} \left| 1-|\beta |\right| \ge \min \{1-f(A-2),f(A)-1,f(-A)+1,-1-f(-A-2)\}. \end{aligned}$$

We compute

$$\begin{aligned} 1-f(A-2)&=\frac{-(A-2)+\sqrt{A^2-4(A-2)}}{2}=\frac{4}{2\left( (A-2)+\sqrt{A^2-4(A-2)}\right) }\\&\ge \frac{2}{A-2+\sqrt{A^2-2A+1}}=\frac{2}{2A-3}, \end{aligned}$$

provided that $A^2-2A+1\ge A^2-4A+8$, which certainly holds for $A\ge 7/2$. Similar computations yield

$$\begin{aligned} f(A)-1\ge \frac{2}{2A-4},\quad f(-A)+1\ge \frac{2}{2A+4},\quad -1-f(-A-2)\ge \frac{2}{2A+5}, \end{aligned}$$

provided that $$A\ge 4$$. $$\square $$

Next, we show that under our assumptions $|\beta |$ is not to large.

### Lemma 7

Assume that $|B|<A^{2-\epsilon }$ and that $A^{\epsilon } \ge 8$. Then we have $\frac{\alpha }{|\beta |}>\frac{1}{2}A^{\epsilon }$.

### Proof

First we note that

$$\begin{aligned} \left| \frac{\alpha }{\beta }\right|&=\left| \frac{A+\sqrt{A^2-4B}}{A-\sqrt{A^2-4B}}\right| =\left| \frac{2A^2-4B+2A\sqrt{A^2-4B}}{4B} \right| \\&\ge \frac{2A^2-4|B|+2A\sqrt{A^2-4|B|}}{4|B|} =\frac{A^2}{2|B|} - 1 + \frac{A^2}{2|B|} \sqrt{1-\frac{4|B|}{A^2}}. \end{aligned}$$

From the observation

$$\begin{aligned} \frac{A^2}{2|B|} \sqrt{1-\frac{4|B|}{A^2}}> \frac{A^{\epsilon }}{2} \sqrt{1-\frac{4}{A^{\epsilon }}} \ge 2\sqrt{2} > 1, \end{aligned}$$

then it follows

$$\begin{aligned} \frac{\alpha }{|\beta |}> \frac{A^2}{2|B|} > \frac{A^{\epsilon }}{2}. \end{aligned}$$

$$\square $$

### Lemma 8

Assume that $|B|<\kappa A$ and that $A \ge 4 \kappa$. Then we have $|\beta |<2\kappa$.

### Proof

We have

$$\begin{aligned} |\beta |=\left| \frac{A-\sqrt{A^2-4B}}{2}\right| \le \frac{2|B|}{A+\sqrt{A^2-4|B|}}. \end{aligned}$$

Let us now consider the function $f(x)=\frac{2x}{A+\sqrt{A^2-4x}}$. This function is strictly increasing with *x* since obviously the numerator is strictly increasing with *x* while the denominator is strictly decreasing with *x*. Therefore we obtain

$$\begin{aligned} |\beta |< \frac{2 \kappa A}{A+\sqrt{A^2-4\kappa A}}=\kappa \frac{2}{1+\sqrt{1-\frac{4\kappa }{A}}}\le 2 \kappa \end{aligned}$$

since $$A\ge 4\kappa $$. $$\square $$

Now let us take a look at the recurrence sequence $V_n$.

### Lemma 9

Assume that $|B|<A^{2-\epsilon }$ and $A^{\epsilon } \ge 8$. Then $V_n$ is strictly increasing for $n\ge \frac{3}{2\epsilon }$.

### Proof

First, we note that

$$\begin{aligned} V_{n+1}-V_n=\alpha ^{n+1}+\beta ^{n+1}-\alpha ^n-\beta ^n=\alpha ^{n}(\alpha -1)+\beta ^{n}(\beta -1)>0 \end{aligned}$$

certainly holds if

$$\begin{aligned} \alpha ^n(\alpha -1)>|\beta |^n(|\beta |+1), \end{aligned}$$

i.e.

5 $$\begin{aligned} \left( \frac{\alpha }{|\beta |}\right) ^n>\frac{|\beta |+1}{\alpha -1}. \end{aligned}$$

Note that $\frac{\alpha }{|\beta |}>\frac{1}{2}A^\epsilon$, by Lemma 7, and, by Lemma 5,

$$\begin{aligned} |\beta |=\frac{|B|}{\alpha }<\frac{2A^{2-\epsilon }}{A}=2 A^{1-\epsilon }. \end{aligned}$$

Moreover, note that the smallest possible value for $\alpha$ is $\frac{1+\sqrt{5}}{2}$. Therefore (5) is certainly fulfilled if

$$\begin{aligned} \left( \frac{1}{2}A^{\epsilon }\right) ^n> 6A^{1-\epsilon } > \frac{3A^{1-\epsilon }}{\frac{-1+\sqrt{5}}{2}} \ge \frac{2A^{1-\epsilon }+1}{\frac{1+\sqrt{5}}{2}-1}. \end{aligned}$$

Thus $V_n$ is increasing if

$$\begin{aligned} n\left( \epsilon \log A - \log 2\right) >\log 6+(1-\epsilon ) \log A \end{aligned}$$

or equivalently

$$\begin{aligned} n>\frac{(1-\epsilon ) \log A+\log 6}{\epsilon \log A-\log 2}. \end{aligned}$$

Since the rational function $f(x)=\frac{ax+b}{cx+d}$ is strictly increasing if $ad-bc>0$, strictly decreasing if $ad-bc<0$, and constant if $ad-bc=0$, we obtain that for

$$\begin{aligned} n \ge \frac{3}{2\epsilon } > \frac{\frac{1-\epsilon }{\epsilon } \log 8 + \log 6}{\log 8 - \log 2} \end{aligned}$$

the Lucas sequence is strictly increasing. $\square$

### Lemma 10

Assume that $|B|<\kappa A$ and $A \ge 4\kappa + 4$. Then $V_n$ is strictly increasing for $n\ge 0$.

### Proof

By (5) we know that $V_n$ is increasing for all $n\ge 0$ if $1>\frac{|\beta |+1}{\alpha -1}$. By Lemma 5, we have $\alpha>\frac{A}{2} \ge 2\kappa +2>2$ and, using Lemma 8, also $|\beta |<2\kappa$. Therefore we get

$$\begin{aligned} \frac{|\beta |+1}{\alpha -1} < \frac{2\kappa +1}{\frac{A}{2}-1} \le 1. \end{aligned}$$

$$\square $$

### Remark 1

Note that assuming $|B|<\kappa A$ and $A\ge \kappa ^2$ implies $|B|<A^{3/2}=A^{2-\epsilon }$ with $\epsilon =1/2$. Therefore all results that are proven under the assumption $|B|<A^{2-\epsilon }$ with $\epsilon =1/2$ also hold under the assumption $|B|<\kappa A$ and $A\ge \kappa ^2$.

In view of this remark and in view of the proofs of the following lemmas, we will assume for the rest of the paper that one of the following two assumptions holds:

1. $$|B|<A^{2-\epsilon }$$, $$A^{\epsilon }\ge 32$$ and $$N_0= \frac{3}{2\epsilon }$$,
2. $$|B|<\kappa A$$, $$A\ge \max \{\kappa ^2,16\kappa +12,1024\}$$ and $$N_0=1$$.

### Remark 2

Let us note that the bound $A\ge \kappa ^2$ and $A\ge 1024$ in assumption A2 results in the useful fact that assumption A2 implies assumption A1 with $\epsilon =\frac{1}{2}$, but with $N_0=1$ instead of $\frac{3}{2\epsilon }$. The assumption $A\ge 16\kappa +12$ is mainly used in the proofs of the Lemmas 12 and 13. Moreover, assumptions A1 and A2 both imply $|B| < A^2$.

In view of the assumptions stated above, an important consequence of Lemmas 9 and 10 is the following:

### Corollary 11

Let assumption A1 or A2 be in force and let us assume that (3) has two solutions $(n_1,m_1)$ and $(n_2,m_2)$ with $n_1>n_2\ge N_0$. Then $m_1>m_2$.

### Lemma 12

Let assumption A1 or A2 be in force and $n\ge N_0$. Then we have $\frac{5}{4} \alpha ^n>V_n>\frac{3}{4} \alpha ^n$.

### Proof

Assume that A1 holds. Then, by Lemma 7, we have

$$\begin{aligned} \left( \frac{|\beta |}{\alpha }\right) ^n<\left( \frac{2}{A^\epsilon }\right) ^n< \frac{1}{4}. \end{aligned}$$

If A2 holds, by Lemma 8, we have

$$\begin{aligned} \left( \frac{|\beta |}{\alpha }\right) ^n<\left( \frac{4\kappa }{A}\right) ^n<\frac{1}{4}. \end{aligned}$$

Therefore in any case we get

$$\begin{aligned} \frac{3}{4} \alpha ^n< \alpha ^n\left( 1-\left( \frac{|\beta |}{\alpha }\right) ^n\right) \le \alpha ^n+\beta ^n=V_n \le \alpha ^n\left( 1+\left( \frac{|\beta |}{\alpha }\right) ^n\right) < \frac{5}{4} \alpha ^n. \end{aligned}$$

$$\square $$

Another lemma that will be used frequently is the following:

### Lemma 13

Let assumption A1 or A2 be in force and assume that Eq. (3) has two solutions $(n_1,m_1)$ and $(n_2,m_2)$ with $n_1>n_2\ge N_0$. Then we have

$$\begin{aligned} \frac{5}{2}> \frac{b^{m_1}}{\alpha ^{n_1}}>\frac{3}{8}. \end{aligned}$$

### Proof

Assuming the existence of two different solutions implies by an application of Lemma 12 the inequality

$$\begin{aligned} \frac{5}{4} \alpha ^{n_1}>V_{n_1}>V_{n_1}-V_{n_2}=b^{m_1}-b^{m_2}= b^{m_1}\left( 1-\frac{1}{b^{m_1-m_2}}\right) \ge \frac{1}{2} b^{m_1}, \end{aligned}$$

which proves the first inequality.

For the second inequality, we apply again Lemma 12 to obtain

$$\begin{aligned} V_{n_1}-V_{n_2}>\frac{3}{4} \alpha ^{n_1}\left( 1-\frac{5}{3\alpha ^{n_1-n_2}}\right) . \end{aligned}$$

Since we assume in any case that $\alpha>\frac{A}{2}>4$, we get $V_{n_1}-V_{n_2}> \frac{3}{8} \alpha ^{n_1}$ and thus

$$\begin{aligned} \frac{3}{8} \alpha ^{n_1}<V_{n_1}-V_{n_2}=b^{m_1}-b^{m_2}< b^{m_1}, \end{aligned}$$

which yields the second inequality. $\square$

Finally, let us remind Carmichael’s theorem [2, Theorem XXIV]:

### Lemma 14

Let *A*, *B* be coprime integers with $A>0$ and $A^2-4B>0$. For any $n\ne 1,3$ there exists a prime^1^*p* such that $p\mid V_n$, but $p\not \mid V_m$ for all $1 \le m<n$, except for the case that $n=6$ and $(A,B)=(1,-1)$.

This lemma can be used to prove the following result:

### Lemma 15

Assume that $c=0$ and $A>1$. Then the Diophantine Eq. (3) has at most one solution (*n*, *m*) with $n\ge 1$.

### Proof

Assume that (3) has two solutions $(n_1,m_1), (n_2,m_2)$ with $n_1>n_2\ge 1$. Then by Carmichael’s primitive divisor theorem (Lemma 14) we deduce that $n_1=3$ and $n_2=1$. Since $V_1=A$ and $V_3=A^3-3AB$ we obtain the system of equations

$$\begin{aligned} A=b^{m_2}, \qquad A^3-3AB=b^{m_1} \end{aligned}$$

which yields

$$\begin{aligned} b^{2m_2}-3B=b^{m_1-m_2}. \end{aligned}$$

That is $b\mid 3B$. Since we assume that $\gcd (A,B)=1$ and $A>1$, we deduce $b\mid 3$ and $b=3$. We also conclude that $3\not \mid B$. By considering 3-adic valuations, this yields $m_1-m_2=1$ since $m_2=0$ would imply $A=1$. Hence we have $B=3^{2m_2-1}-1$.

Note that we also assume $A^2-4B>0$ which implies that

$$\begin{aligned} 3^{2m_2}-4 (3^{2m_2-1}-1)= 4-3^{2m_2-1}>0 \end{aligned}$$

holds. But this is only possible for $m_2=1$ and we conclude that $A=3$ and $B=2$. Since $X^2-3X+2=(X-2)(X-1)$ is not irreducible, this is not an admissible case. Therefore there exists at most one solution to (3). $\square$

## Lower bounds for linear forms in logarithms

The main tool in proving our main theorems are lower bounds for linear forms in logarithms of algebraic numbers. In particular, we will use Matveev’s lower bound proven in [7]. Therefore let $\eta \ne 0$ be an algebraic number of degree *d* and let

$$\begin{aligned} a_d\left( X-\eta ^{(1)}\right) \cdots \left( X-\eta ^{(d)}\right) \in \mathbb {Z}[X] \end{aligned}$$

be the minimal polynomial of $\eta$. Then the absolute logarithmic Weil height is defined by

$$\begin{aligned} h(\eta )=\frac{1}{d} \left( \log |a_d|+\sum _{i=1}^{d} \max \{0,\log |\eta ^{(i)}|\}\right) . \end{aligned}$$

With this notation, the following result due to Matveev [7] holds:

### Theorem 16

(Theorem 2.2 with $r=1$ in [7]) Denote by $\eta _1, \ldots , \eta _N$ algebraic numbers, neither 0 nor 1, by $\log \eta _1, \ldots , \log \eta _N$ determinations of their logarithms, by *D* the degree over $\mathbb {Q}$ of the number field $K = \mathbb {Q}(\eta _1,\ldots ,\eta _N)$, and by $b_1, \ldots , b_N$ rational integers with $b_N\ne 0$. Furthermore let $\kappa =1$ if *K* is real and $\kappa =2$ otherwise. For all integers *j* with $1\le j\le N$ choose

$$\begin{aligned} A_j\ge \max \{D h(\eta _j), |\log \eta _j|, 0.16\}, \end{aligned}$$

and set

$$\begin{aligned} E=\max \left\{ \frac{|b_j| A_j}{A_N}\,:\, 1\le j \le N \right\} . \end{aligned}$$

Assume that

$$\begin{aligned} \Lambda :=b_1\log \eta _1+\cdots +b_N \log \eta _N \ne 0. \end{aligned}$$

Then

$$\begin{aligned} \log |\Lambda | \ge -C(N,\kappa )\max \{1,N/6\} C_0 W_0 D^2 \Omega \end{aligned}$$

with

$$\begin{aligned} \Omega =A_1\cdots A_N, \\ C(N,\kappa )= \frac{16}{N!\, \kappa } e^N (2N +1+2 \kappa )(N+2) (4(N+1))^{N+1} \left( \frac{1}{2} eN\right) ^{\kappa }, \\ C_0= \log \left( e^{4.4N+7}N^{5.5}D^2 \log (eD)\right) , \quad W_0=\log (1.5eED \log (eD)). \end{aligned}$$

In our applications, we will be in the situation $N \in \{2,3\}$ and $K=\mathbb {Q}(\alpha )\subseteq \mathbb {R}$, i.e. we have $D=2$ and $\kappa =1$. In this special case Matveev’s lower bounds take the following form:

### Corollary 17

Let the notations and assumptions of Theorem 16 be in force. Furthermore, assume that *K* is a real quadratic number field and that $\Lambda \ne 0$. Then we have

6 $$\begin{aligned} \begin{aligned} \log |\Lambda |&\ge - 7.26\cdot 10^{10}\log (13.81E)\Omega&\qquad \hbox { for}\ N=3,\\ \log |\Lambda |&\ge - 6.7\cdot 10^{8} \log (13.81E)\Omega&\qquad \hbox { for}\ N=2.\\ \end{aligned} \end{aligned}$$

### Remark 3

Let us note that the form of *E* is essential in our proof to obtain an absolute bound for $n_1$ in Theorem 1. Let us also note that in the case of $N=2$ one could use the results of Laurent [6] to obtain numerically better values but with an $\log (E)^2$ term instead. This would lead to numerically smaller upper bounds for concrete applications of our theorems. However, we refrain from the application of these results to keep our long and technical proof more concise.

In order to apply Matveev’s lower bounds, we provide some height computations. First, we note the following well known properties of the absolute logarithmic height (see for example [12, Chapter 3] for a detailed reference):

### Lemma 18

For any $\eta , \gamma \in \overline{\mathbb {Q}}$ and $l \in \mathbb {Q}$ we have

$$\begin{aligned} h(\eta \pm \gamma )&\le h(\eta ) + h(\gamma ) + \log 2, \\ h(\eta \gamma )&\le h(\eta ) + h(\gamma ),\\ h(\eta ^l)&= |l| h(\eta ). \end{aligned}$$

### Remark 4

Note that for a positive integer *b* we have $h(b)=\log b$ and, with $\alpha$ and $\beta$ from Sect. 3, we have

$$\begin{aligned} h(\alpha )=\frac{1}{2} \left( \max \{0,\log \alpha \}+\max \{0,\log |\beta |\}\}\right) \le \log \alpha . \end{aligned}$$

This together with the above mentioned properties yields for $t\in \mathbb {Z}_{>0}$ the bound

$$\begin{aligned} h(\alpha ^t\pm 1) \le t \log \alpha + \log 2. \end{aligned}$$

One other important aspect in applying Matveev’s result (Theorem 16) is that the linear form $\Lambda$ should not vanish. We will resolve this issue with the following lemma:

### Lemma 19

Assume that the Diophantine Eq. (3) has three solutions $(n_1,m_1)$, $(n_2,m_2)$, $(n_3,m_3)\in \mathbb {N}^2$ with $n_1>n_2>n_3>0$. Then we have

$$\begin{aligned} \Lambda _i&:=n_i\log \alpha -m_i\log b\ne 0,&\hbox { for}\ i=1,2,3\\ \Lambda '&:=n_2\log \alpha -m_2\log b+\log \left( \frac{\alpha ^{n_1-n_2}-1}{b^{m_1-m_2}-1}\right) \ne 0, \end{aligned}$$

### Proof

Assume that $\Lambda _i=n_i\log \alpha -m_i\log b=0$ for some $i\in \{1,2,3\}$. But $\Lambda _i=0$ implies $\alpha ^{n_i}-b^{m_i}=0$ which results in view of (3) in

$$\begin{aligned} \alpha ^{n_i}+\beta ^{n_i}-b^{m_i}=\beta ^{n_i}=c. \end{aligned}$$

Since $X^2-AX+B$ is irreducible, $\alpha$ and $\beta$ are Galois conjugates. Therefore, by applying the non-trivial automorphism of $K=\mathbb {Q}(\alpha )$ to the equation $\beta ^{n_i}=c$, we obtain $\alpha ^{n_i}=c$ since $c\in \mathbb {Q}$. But this implies $\beta ^{n_i}=\alpha ^{n_i}$, hence $|\alpha |=|\beta |$, a contradiction to our assumptions.

Now, let us assume that

$$\begin{aligned} \Lambda '=n_2\log \alpha -m_2\log b+\log \left( \frac{\alpha ^{n_1-n_2}-1}{b^{m_1-m_2}-1}\right) = 0. \end{aligned}$$

This implies $\alpha ^{n_1}-\alpha ^{n_2}=b^{m_1}-b^{m_2}$ which results in view of (3) in $\beta ^{n_1}=\beta ^{n_2}$. But then $\beta =0$ or $\beta$ is a root of unity. Both cases contradict our assumption that $X^2-AX+B$ is irreducible and $\beta \in \mathbb {R}$. $\square$

Finally, we want to record three further elementary lemmas that will be helpful. The first lemma is a standard fact from real analysis.

### Lemma 20

If $|x|\le \frac{1}{2}$, then we have $\left| \log (1+x)\right| \le 2|x|$ and

$$\begin{aligned} \frac{2}{9} x^2 \le x-\log (1+x) \le 2 x^2. \end{aligned}$$

### Proof

A direct application of Taylor’s theorem with a Cauchy and Lagrange remainder, respectively. $\square$

Next, we want to state another estimate from real analysis:

### Lemma 21

Let $x\in \mathbb {R}$ and $n \in \mathbb {N}$ such that $|2nx|<\frac{1}{2}$ and $n\ge 1$. Then we have

$$\begin{aligned} \left| (1+x)^n-1\right| \le e^{1/4}n|x|. \end{aligned}$$

### Proof

Since the case $x=0$ is trivial, we may assume $x \ne 0$. From the mean value theorem, we then get

$$\begin{aligned} \frac{\left| (1+x)^n-1 \right| }{|x|} \le n (1+|x|)^{n-1} \le e^{1/4}n. \end{aligned}$$

$$\square $$

The third lemma is due to Pethő and de Weger [8].

### Lemma 22

Let $a,b \ge 0$, $h \ge 1$ and $x\in \mathbb {R}$ be the largest solution of $x=a + b(\log x)^h$. If $b > (e^2/h)^h$, then

$$\begin{aligned} x<2^h\left( a^{1/h}+b^{1/h}\log (h^h b)\right) ^h, \end{aligned}$$

and if $b\le (e^2/h)^h$, then

$$\begin{aligned} x\le 2^h\left( a^{1/h}+2e^2 \right) ^h. \end{aligned}$$

A proof of this lemma can be found in [11, Appendix B].

## A lower bound for |*c*| in terms of $n_1$ and $\alpha$

The purpose of this section is to prove a lower bound for |*c*|. In particular, we prove the following proposition:

### Proposition 23

Assume that assumption A1 or A2 holds and assume that Diophantine Eq. (3) has two solutions $(n_1,m_1)$ and $(n_2,m_2)$ with $n_1>n_2\ge N_0$. Then we have

$$\begin{aligned} |c|>\alpha ^{n_1-K_0\log (27.62 n_1)}-|\beta |^{n_1} \end{aligned}$$

with $$K_0=2.69 \cdot 10^9 \log b$$.

### Proof

Let us first take a look at the case

$$\begin{aligned} \frac{|c|+|\beta |^{n_1}}{\alpha ^{n_1}} \ge \frac{1}{2}. \end{aligned}$$

Here we get immediately

$$\begin{aligned} |c|+|\beta |^{n_1} \ge \frac{1}{2} \alpha ^{n_1} > \alpha ^{n_1-1} \end{aligned}$$

and are done. Now we can assume

$$\begin{aligned} \frac{|c|+|\beta |^{n_1}}{\alpha ^{n_1}} < \frac{1}{2}. \end{aligned}$$

We consider equation

$$\begin{aligned} \alpha ^{n_1}+\beta ^{n_1}-b^{m_1}=c \end{aligned}$$

and obtain

$$\begin{aligned} \left| \frac{b^{m_1}}{\alpha ^{n_1}}-1\right| \le \frac{|c|+|\beta |^{n_1}}{\alpha ^{n_1}}<\frac{1}{2} \end{aligned}$$

which yields

$$\begin{aligned} \left| m_1\log b-n_1\log \alpha \right| \le 2\frac{|c|+|\beta |^{n_1}}{\alpha ^{n_1}}. \end{aligned}$$

The goal is to apply Matveev’s theorem (Theorem 16) with $N=2$. Note that with $\eta _1=b$ and $\eta _2=\alpha$ we choose $A_1=2\log b$ and $A_2=2\log \alpha$, in view of Remark 4, and obtain

$$\begin{aligned} E=\max \left\{ \frac{m_1\log b}{\log \alpha },n_1\right\} . \end{aligned}$$

Due to Lemma 13 we have

$$\begin{aligned} \frac{m_1\log b}{\log \alpha }<n_1+\frac{\log (5/2)}{\log \alpha }<2n_1. \end{aligned}$$

Therefore we obtain by Corollary 17 that

$$\begin{aligned} 2.68 \cdot 10^9 \log b\log \alpha \log (27.62 n_1)&\ge -\log \left| m_1\log b-n_1\log \alpha \right| \\&\ge n_1\log \alpha - \log (|c|+|\beta |^{n_1}) -\log 2 \end{aligned}$$

which implies the content of the proposition. $\square$

## Bounds for $n_1$ in terms of $\log \alpha$

In this section, we will assume that assumption A1 or A2 holds. However, in the proofs we will mainly consider the case that assumption A1 holds. Note that this is not a real restriction since assumption A2 implies assumption A1 with $\epsilon =\frac{1}{2}$ and $N_0=1$ instead of $N_0=\frac{3}{2\epsilon }$ (cf. Remark 2). Also assume that Diophantine Eq. (3) has three solutions $(n_1,m_1)$, $(n_2,m_2)$, $(n_3,m_3)$ with $n_1>n_2>n_3\ge N_0$. In this section we follow the approach of Chim et al. [3] and prove upper bounds for $n_1$ in terms of $\alpha$. To obtain explicit bounds and to keep track of the dependence on $\log b$ and $\log \alpha$ of the bounds we repeat their proof. This section also delivers the set up for the later sections which provide proofs of our main theorems. Moreover, note that the assumption that three solutions exist, simplifies the proof of Chim et al. [3].

The main result of this section is the following statement:

### Proposition 24

Assume that assumption A1 or A2 holds and that Diophantine Eq. (3) has three solutions $(n_1,m_1)$, $(n_2,m_2)$, $(n_3,m_3)$ with $n_1>n_2>n_3\ge N_0$. Then we have

$$\begin{aligned} n_1<2.58\cdot 10^{22} \frac{\log \alpha }{\epsilon } (\log b)^2 \left( \log \left( 7.12\cdot 10^{23}\frac{\log \alpha }{\epsilon } (\log b)^2\right) \right) ^2, \end{aligned}$$

where we choose $\epsilon =1/2$ in case that assumption A2 holds.

Since we assume the existence of two solutions, we have

$$\begin{aligned} V_{n_1}-b^{m_1}=c=V_{n_2}-b^{m_2} \end{aligned}$$

and therefore obtain

7 $$\begin{aligned} \alpha ^{n_1}+\beta ^{n_1}-\alpha ^{n_2}-\beta ^{n_2}=b^{m_1}-b^{m_2}. \end{aligned}$$

Let us write $\gamma :=\min \{\alpha ,\alpha /|\beta |\}$. Note that we have $\gamma>\frac{1}{2} A^\epsilon >\frac{1}{4} \alpha ^\epsilon$, by Lemma 7 and Lemma 5. With this notation we get the inequality

$$\begin{aligned} \left| \frac{b^{m_1}}{\alpha ^{n_1}}-1\right| \le \alpha ^{n_2-n_1}+\frac{b^{m_2}}{\alpha ^{n_1}}+\frac{|\beta |^{n_1}+|\beta |^{n_2}}{\alpha ^{n_1}}. \end{aligned}$$

Note that, depending on whether $|\beta |>1$ or $|\beta |\le 1$, we have

$$\begin{aligned} \frac{|\beta |^{n_1}+|\beta |^{n_2}}{\alpha ^{n_1}}\le {\left\{ \begin{array}{ll} \dfrac{2}{\alpha ^{n_1}} \le 2\gamma ^{-n_1} &{}\quad \hbox { if}\ |\beta |\le 1 \\ 2 \cdot \dfrac{|\beta |^{n_1}}{\alpha ^{n_1}} \le 2\gamma ^{-n_1} &{}\quad \hbox { if}\ |\beta |> 1 \end{array}\right. }. \end{aligned}$$

Therefore, using Lemma 13, we obtain

8 $$\begin{aligned} \begin{aligned} \left| \frac{b^{m_1}}{\alpha ^{n_1}}-1\right|&\le \alpha ^{n_2-n_1}+\frac{b^{m_2}}{\alpha ^{n_1}}+2\gamma ^{-n_1}\\&\le 2\max \left\{ \frac{7}{2} \alpha ^{n_2-n_1},2\gamma ^{-n_1}\right\} \\&\le 7\max \left\{ \alpha ^{n_2-n_1},\gamma ^{-n_1}\right\} . \end{aligned} \end{aligned}$$

First, let us assume that the maximum in (8) is $\gamma ^{-n_1}$. Under our assumptions, we have $A^{\epsilon }\ge 32$ and $n_1\ge 3$, which implies $7\gamma ^{-n_1}<\frac{1}{2}$. Thus taking logarithms and applying Lemma 20 yields

$$\begin{aligned} |\underbrace{m_1\log b-n_1\log \alpha }_{=:\Lambda }| \le 14\gamma ^{-n_1}. \end{aligned}$$

We apply Matveev’s theorem (Theorem 16) with $N=2$. Note that with $\eta _1=b$ and $\eta _2=\alpha$ we choose $A_1=2\log b$ and $A_2=2\log \alpha$, in view of Remark 4, to obtain

$$\begin{aligned} E=\max \left\{ \frac{m_1\log b}{\log \alpha },n_1\right\} . \end{aligned}$$

Note that, due to Lemma 13,

$$\begin{aligned} \frac{m_1\log b}{\log \alpha }<n_1+\frac{\log (5/2)}{\log \alpha }<2n_1. \end{aligned}$$

Therefore we obtain from Corollary 17 that

$$\begin{aligned} 2.68 \cdot 10^9 \log b\log \alpha \log (27.62 n_1)&\ge -\log |\Lambda | \ge n_1 \log \gamma -\log 14 \\&\ge n_1 (\epsilon \log \alpha - \log 4) -\log 14 \\&\ge n_1 \frac{\epsilon }{2} \log \alpha -\log 14, \end{aligned}$$

where for the last inequality we used $A^{\epsilon } \ge 32$. Thus we have

$$\begin{aligned} 5.38 \cdot 10^9 \frac{\log b}{\epsilon } \log (27.62 n_1)>n_1, \end{aligned}$$

which, using Lemma 22, proves Proposition 24 in this case. Note that this also proves, in this specific case, Theorem 1.

Now we assume that the maximum in (8) is $\alpha ^{n_2-n_1}$. By our assumptions on *A* we have $7\alpha ^{n_2-n_1}<\frac{1}{2}$ and obtain, by Lemma 20,

$$\begin{aligned} |\underbrace{m_1\log b-n_1\log \alpha }_{=:\Lambda }|\le 14\alpha ^{n_2-n_1}. \end{aligned}$$

As computed before, an application of Matveev’s theorem yields

$$\begin{aligned} 2.68 \cdot 10^9 \log b\log \alpha \log (27.62 n_1) \ge -\log |\Lambda |\ge (n_1-n_2) \log \alpha -\log 14 \end{aligned}$$

and therefore

9 $$\begin{aligned} 2.69 \cdot 10^9 \log b \log (27.62 n_1)>n_1-n_2. \end{aligned}$$

For the rest of the proof of Proposition 24, we will assume that (9) holds. Since we assume that a third solution exists, the statement of Lemma 13 also holds for $m_2$ and $n_2$ instead of $m_1$ and $n_1$. In particular we have

$$\begin{aligned} m_1&< n_1 \frac{\log \alpha }{\log b} + \frac{\log \frac{5}{2}}{\log b} \le n_1 \frac{\log \alpha }{\log b} + 2\log \frac{5}{2} \\ m_2&> n_2 \frac{\log \alpha }{\log b} + \frac{\log \frac{3}{8}}{\log b} \ge n_2 \frac{\log \alpha }{\log b} + 2\log \frac{3}{8} \end{aligned}$$

which yields

10 $$\begin{aligned} m_1-m_2< \frac{\log \alpha }{\log b}(n_1-n_2)+\log 49< 2.7 \cdot 10^9 \log \alpha \log (27.62 n_1). \end{aligned}$$

Let us rewrite Eq. (7) again to obtain the inequality

$$\begin{aligned} \left| \frac{b^{m_2}}{\alpha ^{n_2}} \cdot \frac{b^{m_1-m_2}-1}{\alpha ^{n_1-n_2}-1}-1\right| \le \frac{|\beta |^{n_1}+|\beta |^{n_2}}{\alpha ^{n_1}-\alpha ^{n_2}} \le 4\gamma ^{-n_1}. \end{aligned}$$

As previously noted we have $4\gamma ^{-n_1}<\frac{1}{2}$ and therefore we obtainWe aim to apply Matveev’s theorem to $\Lambda '$ with $\eta _3=\frac{b^{m_1-m_2}-1}{\alpha ^{n_1-n_2}-1}$. Note that, due to Remark 4 and the properties of heights, we obtain

$$\begin{aligned} \max \{D h(\eta _3), \left| \log \eta _3\right| , 0.16\}&\le 2(m_1-m_2+1)\log b+2(n_1-n_2+1)\log \alpha \\&\le 1.1\cdot 10^{10} \log b\log \alpha \log (27.62 n_1) =:A_3. \end{aligned}$$

Thus we obtain $E\le 2n_1$ as before, and from Matveev’s theorem

$$\begin{aligned} 3.2\cdot 10^{21} (\log b\log \alpha \log (27.62 n_1))^2 \ge n_1 \log \gamma - \log 8 \ge n_1 \frac{\epsilon }{2} \log \alpha -\log 8, \end{aligned}$$

which yields

11 $$\begin{aligned} 6.42\cdot 10^{21} \frac{\log \alpha }{\epsilon } (\log b \log (27.62 n_1))^2>n_1. \end{aligned}$$

If we put $n'=27.62 n_1$ and apply Lemma 22 to (11), then we end up with

$$\begin{aligned} n'< 7.12\cdot 10^{23} \frac{\log \alpha }{\epsilon } (\log b)^2 \left( \log \left( 7.12\cdot 10^{23} \frac{\log \alpha }{\epsilon }(\log b)^2\right) \right) ^2 \end{aligned}$$

which yields the content of Proposition 24.

## Combining linear forms of logarithms

As done before, let us assume that Diophantine Eq. (3) has three solutions $(n_1,m_1)$, $(n_2,m_2)$, $(n_3,m_3)$ with $n_1>n_2>n_3\ge N_0$. Again we assume that assumption A1 or A2 holds.

Let us reconsider inequality (8) with $n_1,m_1,n_2,m_2$ replaced by $n_2,m_2,n_3,m_3$, respectively. Then we obtain

12 $$\begin{aligned} \begin{aligned} \left| \frac{b^{m_2}}{\alpha ^{n_2}}-1\right|&\le \alpha ^{n_3-n_2}+\frac{b^{m_3}}{\alpha ^{n_2}}+2\gamma ^{-n_2}\\&\le 3 \max \left\{ \alpha ^{n_3-n_2},\frac{5}{2}b^{m_3-m_2},2\gamma ^{-n_2}\right\} . \end{aligned} \end{aligned}$$

Let us assume for the next paragraphs that

13 $$\begin{aligned} M_0:=\max \left\{ 3\alpha ^{n_3-n_2},\frac{15}{2}b^{m_3-m_2},6\gamma ^{-n_2}, 7\alpha ^{n_2-n_1},4\gamma ^{-n_1}\right\} <\frac{1}{2}. \end{aligned}$$

Then, by applying Lemma 20 to (8) and (12), we obtain the system of inequalities

$$\begin{aligned} |\underbrace{m_1\log b-n_1\log \alpha }_{=:\Lambda _1}|&\le \max \left\{ 14 \alpha ^{n_2-n_1},8\gamma ^{-n_1}\right\} ,\\ |\underbrace{m_2\log b-n_2\log \alpha }_{=:\Lambda _2}|&\le \max \left\{ 6\alpha ^{n_3-n_2},15b^{m_3-m_2},12\gamma ^{-n_2}\right\} . \end{aligned}$$

We eliminate the term $\log \alpha$ from these inequalities by considering $\Lambda _0=n_2\Lambda _1-n_1\Lambda _2$ and obtain the inequality

14 $$\begin{aligned} \begin{aligned} |\Lambda _0|&=|(n_2m_1-n_1m_2)\log b|\\&\le \max \left\{ 12n_1 \alpha ^{n_3-n_2},30 n_1 b^{m_3-m_2},24 n_1 \gamma ^{-n_2}, 28 n_2\alpha ^{n_2-n_1},16 n_2\gamma ^{-n_1} \right\} . \end{aligned} \end{aligned}$$

Let us write *M* for the maximum on the right hand side of (14). If $n_2m_1-n_1m_2\ne 0$, we obtain the inequality $\log b \le M$. Since we will study the case that $n_2m_1-n_1m_2=0$ in Sect. 8, we will assume for the rest of this section that $n_2m_1-n_1m_2\ne 0$, i.e. we have $\log b \le M$. Therefore we have to consider five different cases. In each case we want to find an upper bound for $\log \alpha$ if possible:

- The case $M=12n_1 \alpha ^{n_3-n_2}\!\!:$ In this case we get
  $$\begin{aligned} \log \log b \le \log (12n_1)-(n_2-n_3)\log \alpha \end{aligned}$$
  which yields
  $$\begin{aligned} \log \alpha \le \log \left( 12n_1/\log b\right) <\log (17.4 n_1), \end{aligned}$$
  since we assume $b\ge 2$.
- The case $M=30 n_1 b^{m_3-m_2}\!\!:$ In this case we obtain
  $$\begin{aligned} \log \log b \le \log (30n_1)-(m_2-m_3)\log b \end{aligned}$$
  which yields
  $$\begin{aligned} m_2-m_3 \le \frac{\log (30n_1/\log b)}{\log b}<1.45 \log (43.3 n_1). \end{aligned}$$
  To obtain from this inequality a bound for $\log \alpha$ is not straight forward and we will deal with this case in Sect. 9.
- The case $M=24 n_1 \gamma ^{-n_2}\!\!:$ This case implies
  $$\begin{aligned} \log \log b \le \log (24 n_1)-n_2\log \gamma \le \log (24 n_1)-n_2 \frac{\epsilon }{2} \log \alpha \end{aligned}$$
  and we obtain
  $$\begin{aligned} \log \alpha \le \frac{2\log \left( 24n_1/\log b\right) }{\epsilon }<\frac{2\log (34.7 n_1)}{\epsilon }. \end{aligned}$$
- The case $M=28 n_2\alpha ^{n_2-n_1}\!\!\!:$ By a similar computation as in the first case, we obtain in this case the inequality
  $$\begin{aligned} \log \alpha<\log (40.4 n_2)< \log (40.4 n_1). \end{aligned}$$
- The case $M=16 n_2\gamma ^{-n_1}\!\!\!:$ Almost the same computations as in the case that $M=24 n_1 \gamma ^{-n_2}$ lead to
  $$\begin{aligned} \log \alpha <\frac{2\log (23.1 n_1)}{\epsilon }. \end{aligned}$$

In the case that (13) does not hold, i.e. that $M_0\ge 1/2$, we obtain by similar computations in each of the five possibilities the following inequalities:

- The case $$M_0=3 \alpha ^{n_3-n_2}\!\!:$$ $$\log \alpha \le \log 6$$;
- The case $$M_0=\frac{15}{2} b^{m_3-m_2}\!\!:$$ $$m_2-m_3\le 3$$;
- The case $$M_0=6 \gamma ^{-n_2}\!\!:$$ $$\log \alpha \le \frac{\log 144}{\epsilon }$$;
- The case $$M_0=7 \alpha ^{n_2-n_1}\!\!:$$ $$\log \alpha \le \log 14$$;
- The case $$M_0=4 \gamma ^{-n_1}\!\!:$$ $$\log \alpha \le \frac{\log 64}{\epsilon }$$.

Let us recap what we have proven so far in the following lemma:

### Lemma 25

Assume that assumption A1 or A2 holds and assume that Diophantine Eq. (3) has three solutions $(n_1,m_1)$, $(n_2,m_2)$, $(n_3,m_3)$ with $n_1>n_2>n_3\ge N_0$. Then one of the following three possibilities holds:

(i) $$n_2m_1-n_1m_2=0$$;
(ii) $$m_2-m_3 < 1.45 \log (43.3 n_1)$$;
(iii) $$\log \alpha <\frac{2\log (34.7 n_1)}{\epsilon }.$$

Since we will deal with the first and second possiblity in the next sections, we close this section by proving that the last possibility implies Theorems 1 and 2. Therefore let us plug in the upper bound for $\log \alpha$ into inequality (11) to obtain

$$\begin{aligned} 1.29\cdot 10^{22} \left( \frac{\log b}{\epsilon }\right) ^2 (\log (34.7 n_1))^3>n_1. \end{aligned}$$

Writing $n'=34.7 n_1$, this inequality turns into

$$\begin{aligned} 4.48\cdot 10^{23} \left( \frac{\log b}{\epsilon }\right) ^2 (\log (n'))^3>n' \end{aligned}$$

and an application of Lemma 22 implies

$$\begin{aligned} n_1 < 1.04 \cdot 10^{23} \left( \frac{\log b}{\epsilon }\right) ^2 \left( \log \left( 1.21 \cdot 10^{25} \left( \frac{\log b}{\epsilon }\right) ^2 \right) \right) ^3. \end{aligned}$$

Thus Theorem 1 is proven in this case.

Now let us assume that assumption A2 holds. By Remark 2 we get the bound

$$\begin{aligned} n_1<4.16 \cdot 10^{23} \left( \log b\right) ^2 \left( \log \left( 4.84 \cdot 10^{25} \left( \log b\right) ^2 \right) \right) ^3. \end{aligned}$$

If we insert our upper bound for $n_1$ into the upper bound for $\log \alpha$, we obtain

$$\begin{aligned} \log A&< \log \alpha + \log 2 \le 5 \log (34.7 n_1) \\&\le 5 \log \left( 1.45 \cdot 10^{25} \left( \log b\right) ^2 \left( \log \left( 4.84 \cdot 10^{25} \left( \log b\right) ^2\right) \right) ^3 \right) . \end{aligned}$$

This proves Theorem 2 in the current case.

## The case $n_1m_2-n_2m_1=0$

We distinguish between the cases $c\ge 0$ and $c<0$.

### The case $c \ge 0$ – bound for $n_1$

In this case we have

15 $$\begin{aligned} 0\le c=V_{n_3}-b^{m_3}<\alpha ^{n_3}+\beta ^{n_3}<2\alpha ^{n_3}. \end{aligned}$$

Furthermore it holds

$$\begin{aligned} \frac{b^{m_1}}{\alpha ^{n_1}}=1+\frac{\beta ^{n_1}-c}{\alpha ^{n_1}}\qquad \text { as well as } \qquad \frac{b^{m_2}}{\alpha ^{n_2}}=1+\frac{\beta ^{n_2}-c}{\alpha ^{n_2}}. \end{aligned}$$

Since

$$\begin{aligned} \left| \frac{\beta ^{n_2}-c}{\alpha ^{n_2}}\right| \le \left( \frac{|\beta |}{\alpha }\right) ^{n_2}+2\alpha ^{n_3-n_2}<\frac{1}{2} \end{aligned}$$

holds under our assumptions, we may apply Lemma 20 to get the two inequalities

$$\begin{aligned} \frac{2}{9} \left( \frac{\beta ^{n_1}-c}{\alpha ^{n_1}} \right) ^2&\le \frac{\beta ^{n_1}-c}{\alpha ^{n_1}} -\left( m_1\log b-n_1\log \alpha \right) \le 2 \left( \frac{\beta ^{n_1}-c}{\alpha ^{n_1}} \right) ^2,\\ \frac{2}{9} \left( \frac{\beta ^{n_2}-c}{\alpha ^{n_2}} \right) ^2&\le \frac{\beta ^{n_2}-c}{\alpha ^{n_2}}-\left( m_2\log b-n_2\log \alpha \right) \le 2 \left( \frac{\beta ^{n_2}-c}{\alpha ^{n_2}} \right) ^2. \end{aligned}$$

Multiplying the first inequality by $n_2$ and the second one by $n_1$ as well as forming the difference afterwards yields

16 $$\begin{aligned} \begin{aligned} \frac{2n_2}{9} \left( \frac{\beta ^{n_1}-c}{\alpha ^{n_1}} \right) ^2-2n_1\left( \frac{\beta ^{n_2}-c}{\alpha ^{n_2}} \right) ^2&\le n_2\frac{\beta ^{n_1}-c}{\alpha ^{n_1}}-n_1\frac{\beta ^{n_2}-c}{\alpha ^{n_2}} \\&\le 2n_2\left( \frac{\beta ^{n_1}-c}{\alpha ^{n_1}} \right) ^2- \frac{2n_1}{9} \left( \frac{\beta ^{n_2}-c}{\alpha ^{n_2}} \right) ^2. \end{aligned} \end{aligned}$$

Let us note that $(a+b)^2\le 4\max \{|a|^2,|b|^2\}$, and therefore we obtain

$$\begin{aligned} c\left( \frac{n_1}{\alpha ^{n_2}}-\frac{n_2}{\alpha ^{n_1}}\right)&\le \frac{n_2|\beta |^{n_1}}{\alpha ^{n_1}}+\frac{n_1|\beta |^{n_2}}{\alpha ^{n_2}}+8n_2\max \left\{ \frac{|\beta |^{2n_1}}{\alpha ^{2n_1}},\frac{c^2}{\alpha ^{2n_1}}\right\} \\&\le 10n_1\max \left\{ \frac{|\beta |^{n_2}}{\alpha ^{n_2}},\frac{c^2}{\alpha ^{2n_2}} \right\} . \end{aligned}$$

Together with the estimate

$$\begin{aligned} \frac{n_1}{\alpha ^{n_2}}-\frac{n_2}{\alpha ^{n_1}}>\frac{n_1}{\alpha ^{n_2}}\left( 1-\frac{1}{\alpha }\right) >\frac{7}{8} \cdot \frac{n_1}{\alpha ^{n_2}} \end{aligned}$$

this implies

17 $$\begin{aligned} c< 12\max \left\{ |\beta |^{n_2},\frac{c^2}{\alpha ^{n_2}}\right\} . \end{aligned}$$

Let us assume for the moment that the maximum is $\frac{c^2}{\alpha ^{n_2}}$. Then we obtain

$$\begin{aligned} \alpha ^{n_2}< 12 c < 24\alpha ^{n_3} \end{aligned}$$

which implies $\alpha <24$ and thus Theorem 2. Plugging in $\alpha <24$ in Proposition 24 yields the content of Theorem 1 in this case.

Therefore we assume now $c<12|\beta |^{n_2}$. By Proposition 23 we obtain

$$\begin{aligned} \alpha ^{n_1-K_0\log (27.62 n_1)}-|\beta |^{n_1}<|c|<12|\beta |^{n_2} \end{aligned}$$

which yields

$$\begin{aligned} \left( n_1-K_0\log (27.62 n_1)\right) \log \alpha <\log 13 +n_1\max \{\log |\beta |,0\}. \end{aligned}$$

Note that, due to our assumptions, we have the bound

$$\begin{aligned} \alpha ^{1-\frac{\epsilon }{4}} \ge 8\alpha ^{1-\epsilon }> 4A^{1-\epsilon }> \frac{2\alpha }{A^{\epsilon }}>|\beta | \end{aligned}$$

which implies $(1-\frac{\epsilon }{4}) \log \alpha > \log |\beta |$. Thus we get

$$\begin{aligned} n_1 \frac{\epsilon }{4} \log \alpha < K_0 \log \alpha \log (27.62 n_1)+\log 13 \end{aligned}$$

and

$$\begin{aligned} n_1< 1.08 \cdot 10^{10} \frac{\log b}{\epsilon } \log (27.62 n_1). \end{aligned}$$

As previously, solving this inequality with the help of Lemma 22 yields

18 $$\begin{aligned} n_1< 2.17 \cdot 10^{10} \frac{\log b}{\epsilon }\log \left( 2.99 \cdot 10^{11} \frac{\log b}{\epsilon } \right) \end{aligned}$$

which proves Theorem 1 in this case.

So we may now assume that assumption A2 holds and the bound for $n_1$ with $\epsilon =\frac{1}{2}$ is valid. This yields

19 $$\begin{aligned} n_1< 4.34 \cdot 10^{10}\log b\log \left( 5.98 \cdot 10^{11} \log b\right) . \end{aligned}$$

### The case $$ c \ge 0 $$$$ - $$ bound for $$ \log A $$

For $c \ge 0$ it remains to prove the bound for $\log A$ stated in our second theorem. We can already use the above proven bound (19) for $n_1$ since we assume assumption A2. Note that under assumption A2 we have $|\beta |<2\kappa$, by Lemma 8. Hence the quantities $|c|,|\beta |^{n_1},|\beta |^{n_2}$ are bounded by effectively computable constants depending only on $\kappa$ and *b*.

Let us consider the case $|c-\beta ^{n_2}|\ge 2(|c|+|\beta |^{n_1})\alpha ^{n_2-n_1}$. Note that $\beta ^{n_2} \ne c$ by the usual Galois conjugation argument. If $c > \beta ^{n_2}$, then (16) gives us

$$\begin{aligned} (2n_1-n_2)\frac{|c|+|\beta |^{n_1}}{\alpha ^{n_1}}&\le n_2\frac{\beta ^{n_1}-c}{\alpha ^{n_1}}-n_1\frac{\beta ^{n_2}-c}{\alpha ^{n_2}} \\&\le 2n_2\left( \frac{\beta ^{n_1}-c}{\alpha ^{n_1}} \right) ^2- \frac{2n_1}{9} \left( \frac{\beta ^{n_2}-c}{\alpha ^{n_2}} \right) ^2 \\&\le 2n_1\left( \frac{|\beta |^{n_1}+|c|}{\alpha ^{n_1}} \right) ^2 \end{aligned}$$

which yields

$$\begin{aligned} \alpha ^{n_1}\le 2(|\beta |^{n_1}+|c|). \end{aligned}$$

As $c<2\alpha ^{n_3}$ (see inequality (15)) we obtain

$$\begin{aligned} 0.2 \alpha ^{n_1}< \alpha ^{n_1}-2c \le 2|\beta |^{n_1} < 2(2\kappa )^{n_1} \end{aligned}$$

which yields $$\alpha < 20 \kappa $$.

If $c < \beta ^{n_2}$, then (16) gives us

$$\begin{aligned} \left( n_1-\frac{n_2}{2}\right) \frac{\beta ^{n_2}-c}{\alpha ^{n_2}}&\le n_1\frac{\beta ^{n_2}-c}{\alpha ^{n_2}} - n_2\frac{\beta ^{n_1}-c}{\alpha ^{n_1}} \\&\le 2n_1\left( \frac{\beta ^{n_2}-c}{\alpha ^{n_2}} \right) ^2- \frac{2n_2}{9} \left( \frac{\beta ^{n_1}-c}{\alpha ^{n_1}} \right) ^2 \le 2n_1\left( \frac{\beta ^{n_2}-c}{\alpha ^{n_2}} \right) ^2 \end{aligned}$$

which implies

$$\begin{aligned} 1 \le 4\cdot \frac{\beta ^{n_2}-c}{\alpha ^{n_2}} \le 4\cdot \frac{|\beta |^{n_2}+|c|}{\alpha ^{n_2}} \le 52\cdot \frac{|\beta |^{n_2}}{\alpha ^{n_2}} \le 52\cdot \frac{|\beta |}{\alpha } < 104 \kappa \alpha ^{-1} \end{aligned}$$

and hence $$ \alpha < 104\kappa $$.

Thus we may now assume $|c-\beta ^{n_2}|< 2(|c|+|\beta |^{n_1})\alpha ^{n_2-n_1}$. Let us note that under the assumption $\alpha >4(|c|+\max \{1,|\beta |\}^{n_1})$ we can deduce

20 $$\begin{aligned} \left| \frac{b^{m_3}}{\alpha ^{n_3}}-1\right| \le \frac{|c|+|\beta |^{n_3}}{\alpha ^{n_3}}<\frac{1}{4}, \end{aligned}$$

and otherwise we would get the constant $C_2$ in Theorem 2 (cf. the calculations below). Then, by Lemma 20, we get

$$\begin{aligned} |\underbrace{m_3\log b-n_3\log \alpha }_{=:\Lambda _3}| \le \frac{2|c|+2|\beta |^{n_3}}{\alpha ^{n_3}} \le \frac{24|\beta |^{n_2}+2|\beta |^{n_3}}{\alpha ^{n_3}} \le \frac{26(2\kappa )^{n_1}}{\alpha ^{n_3}}. \end{aligned}$$

Recalling from the beginning of Sect. 7 the bound

$$\begin{aligned} |\underbrace{m_1\log b-n_1\log \alpha }_{=:\Lambda _1}| \le \max \left\{ 14 \alpha ^{n_2-n_1},8\gamma ^{-n_1}\right\} , \end{aligned}$$

we can again eliminate the term $\log \alpha$ from these inequalities by considering the form $\Lambda _0'=n_3\Lambda _1-n_1\Lambda _3$ and obtain the inequality

21 $$\begin{aligned} \begin{aligned} |\Lambda _0'|&=|(n_3m_1-n_1m_3)\log b|\\&\le \max \left\{ 52n_1(2\kappa )^{n_1} \alpha ^{-n_3}, 28 n_3\alpha ^{n_2-n_1},16 n_3\gamma ^{-n_1} \right\} \\&\le \max \left\{ 52n_1(2\kappa )^{n_1} \alpha ^{-n_3}, 28 n_3\alpha ^{n_2-n_1},16 n_3\alpha ^{-n_1/4} \right\} . \end{aligned} \end{aligned}$$

If $n_3m_1-n_1m_3 \ne 0$, then we have

$$\begin{aligned} \log b \le \max \left\{ 52n_1(2\kappa )^{n_1} \alpha ^{-n_3}, 28 n_3\alpha ^{n_2-n_1},16 n_3\alpha ^{-n_1/4} \right\} \end{aligned}$$

which yields $\log \alpha \le 5 + \log n_1 + n_1 \log (4\kappa )$, and together with the bound (19) this gives us constant $C_2$ in Theorem 2.

Hence we can assume $n_3m_1-n_1m_3 = 0$ and replace in the discussion above $m_2$ by $m_3$ as well as $n_2$ by $n_3$. Since by (20) we have

$$\begin{aligned} \left| \frac{\beta ^{n_3}-c}{\alpha ^{n_3}} \right| = \left| \frac{b^{m_3}}{\alpha ^{n_3}}-1\right| < \frac{1}{4}, \end{aligned}$$

we may apply Lemma 20 also to this expression and get an analogous version of (16) with $n_2$ replaced by $n_3$. The consideration of $|c-\beta ^{n_3}| \ge 2(|c|+|\beta |^{n_1})\alpha ^{n_3-n_1}$ yields in the case $c > \beta ^{n_3}$, in the same way as above, $\alpha < 20\kappa$, and in the case $c < \beta ^{n_3}$ we apply the analogous version of (16), as done above, with the conclusion

$$\begin{aligned} 1 \le 4 \cdot \frac{\beta ^{n_3}-c}{\alpha ^{n_3}} \le 4 \cdot \left| \frac{\beta ^{n_3}-c}{\alpha ^{n_3}} \right| < 1, \end{aligned}$$

a contradiction stating that this case is not possible. For this reason we may now assume $|c-\beta ^{n_3}|< 2(|c|+|\beta |^{n_1})\alpha ^{n_3-n_1}$. Thus altogether we obtain

22 $$\begin{aligned} |\beta |^{n_3}\left| \beta ^{n_2-n_3}-1\right| =\left| \beta ^{n_2}-\beta ^{n_3}\right| <4(|c|+|\beta |^{n_1})\alpha ^{n_2-n_1}. \end{aligned}$$

From (22) we deduce that one of the two factors of the left hand side is smaller than $2\alpha ^{\frac{n_2-n_1}{2}}\sqrt{|c|+|\beta |^{n_1}}$. By thinking of constant $C_2$ in Theorem 2, we may assume $\alpha >64(|c| +|\beta |^{n_1})$. So we have $2\alpha ^{\frac{n_2-n_1}{2}}\sqrt{|c|+|\beta |^{n_1}} < \frac{1}{4}$. Let us first assume that

$$\begin{aligned} |\beta |^{n_3} < 2\alpha ^{\frac{n_2-n_1}{2}}\sqrt{|c|+|\beta |^{n_1}}. \end{aligned}$$

This implies $|\beta |^{n_3}< \frac{1}{4}$ and further

$$\begin{aligned} |c|< |\beta |^{n_3}+2(|c|+|\beta |^{n_1})\alpha ^{n_3-n_1}<\frac{1}{4} +\frac{1}{8}<1. \end{aligned}$$

Therefore we have $c=0$. But Lemma 15 states that there cannot be three solutions for $c=0$.

Now we may assume

$$\begin{aligned} \left| \beta ^{n_2-n_3}-1\right| < 2\alpha ^{\frac{n_2-n_1}{2}}\sqrt{|c|+|\beta |^{n_1}}. \end{aligned}$$

Here we get the further bound

$$\begin{aligned} ||\beta |-1|\le \left| |\beta |^{n_2-n_3}-1\right| \le \left| \beta ^{n_2-n_3}-1\right| < 2\alpha ^{\frac{n_2-n_1}{2}}\sqrt{|c|+|\beta |^{n_1}}. \end{aligned}$$

Assuming $\alpha >64n_2^2(|c|+|\beta |^{n_1})$, we obtain by an application of Lemma 21 that

$$\begin{aligned} ||\beta |^{n_2}-1| \le 2.6 n_2 \alpha ^{\frac{n_2-n_1}{2}}\sqrt{|c|+|\beta |^{n_1}}. \end{aligned}$$

This together with our assumption $||c|-|\beta |^{n_2}| \le |c-\beta ^{n_2}|< 2(|c|+|\beta |^{n_1})\alpha ^{n_2-n_1}$ gives us

$$\begin{aligned} ||c|-1|<2.6 n_2 \alpha ^{\frac{n_2-n_1}{2}}\sqrt{|c|+|\beta |^{n_1}}+ 2(|c|+|\beta |^{n_1})\alpha ^{n_2-n_1}<\frac{1}{2} \end{aligned}$$

provided that

$$\begin{aligned} \alpha \ge 109 n_2^2(|c|+|\beta |^{n_1}). \end{aligned}$$

Thus we may assume $c=1$ provided that $\alpha$ is large enough. But this also implies

$$\begin{aligned} |1-\beta ^{n_3}|<2(1+|\beta |^{n_1})\alpha ^{n_3-n_1}\le 2(1+|\beta |^{n_1})\alpha ^{-2}. \end{aligned}$$

If $\beta ^{n_3}<0$, we get

$$\begin{aligned} \alpha <\sqrt{2(1+|\beta |^{n_1})} \le 2 (|c|+|\beta |^{n_1}). \end{aligned}$$

Therefore we may assume $\beta ^{n_3}=|\beta |^{n_3}$ is positive. Since for any real numbers $x>0$ and $n\ge 1$ we have $|1-x| \le |1-x^n|$, we obtain from Lemma 6 together with Lemma 5

$$\begin{aligned} \frac{2}{4\alpha +5}<\frac{2}{2A+5}\le |1-|\beta ||\le |1-\beta ^{n_3}|<2(|c|+|\beta |^{n_1})\alpha ^{-2}. \end{aligned}$$

Hence we get

$$\begin{aligned} \alpha <9(|c|+|\beta |^{n_1}) \end{aligned}$$

in this case.

So it remains to consider the situation

$$\begin{aligned} \alpha< 109 n_2^2(|c|+|\beta |^{n_1}) < 1417 n_1^2(2\kappa )^{n_1}. \end{aligned}$$

With (19) and Lemma 5 this implies

$$\begin{aligned} \log A&<n_1\log (4\kappa )+2\log n_1+\log 2834\\&< 4.35 \cdot 10^{10}\log (4\kappa ) \log b\log \left( 5.98 \cdot 10^{11} \log b\right) \end{aligned}$$

and Theorem 2 is proven in that case.

### The case $c< 0$

The case $c<0$ can be treated with similar arguments. Therefore we will only point out the differences.

We start with the observation

$$\begin{aligned} 0<|c|=-c=b^{m_3}-V_{n_3}<b^{m_3} \end{aligned}$$

and write again

$$\begin{aligned} \frac{b^{m_1}}{\alpha ^{n_1}}=1+\frac{\beta ^{n_1}-c}{\alpha ^{n_1}}\qquad \text { as well as } \qquad \frac{b^{m_2}}{\alpha ^{n_2}}=1+\frac{\beta ^{n_2}-c}{\alpha ^{n_2}}. \end{aligned}$$

Note that, using Lemma 13,

$$\begin{aligned} \left| \frac{\beta ^{n_2}-c}{\alpha ^{n_2}}\right| \le \left( \frac{|\beta |}{\alpha }\right) ^{n_2}+\frac{|c|}{\alpha ^{n_2}}< \frac{1}{16} + \frac{b^{m_3}}{\alpha ^{n_2}}< \frac{1}{16} + \frac{5}{2} b^{m_3-m_2} < \frac{1}{2} \end{aligned}$$

holds under our assumptions if in addition $m_2-m_3 \ge 2$. The case $m_2-m_3 =1$ is included in the next section. Thus we get again the inequality chain (16) and furthermore the bound

$$\begin{aligned} |c|< 12\max \left\{ |\beta |^{n_2},\frac{|c|^2}{\alpha ^{n_2}}\right\} . \end{aligned}$$

If the maximum is $\frac{|c|^2}{\alpha ^{n_2}}$, then we have

$$\begin{aligned} \frac{2}{5} b^{m_2}< \alpha ^{n_2}< 12|c| < 12 b^{m_3} \end{aligned}$$

which implies $m_2-m_3 \le 3$. This will be handled in Sect. 9. Therefore we may now again assume $|c| < 12|\beta |^{n_2}$. In the same way as in the case $c \ge 0$ we obtain again the upper bound (18) proving Theorem 1 also in the case $c<0$. Moreover, we get under assumption A2 the same upper bound (19) for $n_1$. In particular, the quantities $|c|,|\beta |^{n_1},|\beta |^{n_2}$ are bounded by effectively computable constants depending only on $\kappa$ and *b*.

The reader might already have noticed that, in the case $c \ge 0$, we sometimes have written |*c*| and sometimes *c*. We did this in order to reuse these calculations now for the case $c<0$. The only adaptions we need for $c<0$ when going through the previous subsection are the following: First, the special case

$$\begin{aligned} \alpha ^{n_1} \le 2(|\beta |^{n_1}+|c|) \end{aligned}$$

now, by Lemma 13, yields

$$\begin{aligned} \frac{1}{15}\alpha ^{n_1}< \frac{8}{45} b^{m_1}< \alpha ^{n_1}-2b^{m_3}< \alpha ^{n_1}-2|c| \le 2|\beta |^{n_1} < 2(2\kappa )^{n_1} \end{aligned}$$

and thus $\alpha < 60 \kappa$. Second, we have to consider $c=-1$ instead of $c=1$, which implies

$$\begin{aligned} |1+\beta ^{n_3}|<2(1+|\beta |^{n_1})\alpha ^{n_3-n_1}\le 2(1+|\beta |^{n_1})\alpha ^{-2}. \end{aligned}$$

If $\beta ^{n_3}>0$, we get

$$\begin{aligned} \alpha <\sqrt{2(1+|\beta |^{n_1})} \le 2 (|c|+|\beta |^{n_1}), \end{aligned}$$

and if $\beta ^{n_3}=-|\beta |^{n_3}$ is negative, we obtain from Lemma 6 together with Lemma 5

$$\begin{aligned} \frac{2}{4\alpha +5}<\frac{2}{2A+5}\le |1-|\beta ||\le |1+\beta ^{n_3}|<2(|c|+|\beta |^{n_1})\alpha ^{-2} \end{aligned}$$

and again

$$\begin{aligned} \alpha <9(|c|+|\beta |^{n_1}). \end{aligned}$$

The other steps work as above. Hence Theorem 2 is proven in this case as well.

Let us summarize what we have proven so far:

#### Lemma 26

Assume that assumption A1 or A2 holds and assume that Diophantine Eq. (3) has three solutions $(n_1,m_1)$, $(n_2,m_2)$, $(n_3,m_3)$ with $n_1>n_2>n_3\ge N_0$. Then at least one of the following three possibilities holds:

1. assumption A1 holds and
  $$\begin{aligned} n_1 < 1.04 \cdot 10^{23} \left( \frac{\log b}{\epsilon }\right) ^2 \left( \log \left( 1.21 \cdot 10^{25} \left( \frac{\log b}{\epsilon }\right) ^2 \right) \right) ^3; \end{aligned}$$
2. assumption A2 holds and
  $$\begin{aligned} \log A < 4.35 \cdot 10^{10}\log (4\kappa ) \log b\log \left( 5.98 \cdot 10^{11} \log b\right) \end{aligned}$$
  or
  $$\begin{aligned} \log A < 5 \log \left( 1.45 \cdot 10^{25} \left( \log b\right) ^2 \left( \log \left( 4.84 \cdot 10^{25} \left( \log b\right) ^2\right) \right) ^3 \right) ; \end{aligned}$$
3. $$m_2-m_3<1.45 \log (43.3 n_1)$$.

## The case $m_2-m_3\ll \log n_1$

In view of Theorems 1 and 2 and Lemma 26 we may assume that assumption A1 or A2 holds and that $m_2-m_3<1.45 \log (43.3 n_1)$.

First, we reconsider inequality (8) and note that $7\max \left\{ \alpha ^{n_2-n_1},\gamma ^{-n_1}\right\} \ge \frac{1}{2}$ implies either $\alpha \le 14$ or $A^{\epsilon } \le 28$. In the first cases we have an upper bound for $\alpha$ and by Proposition 24 also an upper bound for $n_1$; the second case contradicts assumption A1 and A2 respectively. Thus Theorems 1 and 2 are shown in those situations. Now we may apply Lemma 20 and obtain, as in Sect. 6, the inequality

23 $$\begin{aligned} |\underbrace{n_1\log \alpha -m_1\log b}_{=:\Lambda _1}| \le 14 \max \left\{ \alpha ^{n_2-n_1},\gamma ^{-n_1}\right\} . \end{aligned}$$

Next, let us consider the inequality

$$\begin{aligned} \left| \frac{\alpha ^{n_2}}{b^{m_2}-b^{m_3}}-1\right| =\left| \frac{\alpha ^{n_2}}{b^{m_3}(b^{m_2-m_3}-1)}-1\right|&\le \frac{\alpha ^{n_3}+|\beta |^{n_2}+|\beta |^{n_3}}{b^{m_2}-b^{m_3}}\\&< 6\alpha ^{n_3-n_2}+12\gamma ^{-n_2}\\&\le 18\max \{\alpha ^{n_3-n_2},\gamma ^{-n_2}\}. \end{aligned}$$

In particular, note that $b^{m_2}-b^{m_3}\ge \frac{1}{2} b^{m_2} > \frac{3}{16} \alpha ^{n_2}$ by Lemma 13. Assuming that $18\alpha ^{n_3-n_2} \ge \frac{1}{2}$ yields $\alpha \le 36$ which implies by Proposition 24 Theorems 1 and 2. Similarly, using $n_2\ge 2$, the assumption $18\gamma ^{-n_2} \ge \frac{1}{2}$ gives us either $\alpha \le 6$ or $A^{\epsilon } \le 12$ and we are done as well. Thus we may apply Lemma 20 and obtain

24 $$\begin{aligned} |\underbrace{n_2\log \alpha -m_3\log b-\log (b^{m_2-m_3}-1)}_{=:\Lambda _2}| \le 36\max \{\alpha ^{n_3-n_2},\gamma ^{-n_2}\}. \end{aligned}$$

Eliminating the term $\log \alpha$ from the linear forms $\Lambda _1$ and $\Lambda _2$ by considering $\Lambda =n_1\Lambda _2-n_2\Lambda _1$ yields together with (23) and (24) the bound

25 $$\begin{aligned} \begin{aligned} |\Lambda |&\le 36n_1\max \{\alpha ^{n_3-n_2},\gamma ^{-n_2}\}+14n_2\max \{\alpha ^{n_2-n_1},\gamma ^{-n_1}\}\\&\le 50n_1\max \{\alpha ^{n_3-n_2},\gamma ^{-n_2},\alpha ^{n_2-n_1}\}\\&\le 200n_1\alpha ^{-\epsilon }, \end{aligned} \end{aligned}$$

where

$$\begin{aligned} \Lambda =(m_1n_2-m_3n_1)\log b-n_1\log (b^{m_2-m_3}-1). \end{aligned}$$

Now we have to distinguish between the cases $\Lambda =0$ and $\Lambda \ne 0$.

### The case $\Lambda = 0$

Since $n_1\ne 0$ this case can only occur if *b* and $b^{m_2-m_3}-1$ are multiplicatively dependent. This is only possible if $b=2$ and $m_2-m_3=1$, i.e. if $b^{m_2-m_3}-1=1$. Note that our assumptions imply $c\ge 0$ if $b=2$. Therefore we obtain

$$\begin{aligned} 0\le c =V_{n_3}-b^{m_3}=\alpha ^{n_3}+\beta ^{n_3}-b^{m_3} \end{aligned}$$

which implies $$b^{m_3}\le 2\alpha ^{n_3}$$.

From Lemma 13 we know that $b^{m_2}>\frac{3}{8} \alpha ^{n_2}$. Hence, using the facts $b=2$ and $m_2=m_3+1$, we get the inequality

$$\begin{aligned} \frac{3}{8} \alpha ^{n_2}<b^{m_2}=2b^{m_3}\le 4\alpha ^{n_3} \end{aligned}$$

which implies $\alpha ^{n_2-n_3}<11$ and thus $\alpha <11$. An application of Proposition 24 yields Theorems 1 and 2.

#### Remark 5

Let us note that in the case $c<0$ the argument above does not work. This is the reason why we exclude $b=2$ if $c<0$.

### The case $\Lambda \ne 0$

Here we may apply Matveev’s theorem, Theorem 16, to $\Lambda$. Note that the case $b^{m_2-m_3}-1=1$ can be excluded by the previous subsection.

First, let us find an upper bound for $|m_1n_2-m_3n_1|$. We deduce from (25) the bound

$$\begin{aligned} |m_1n_2-m_3n_1|\log b&\le n_1\log (b^{m_2-m_3}-1) + 200n_1 \\&\le n_1(m_2-m_3)\log b + 200n_1 \end{aligned}$$

which implies

$$\begin{aligned} |m_1n_2-m_3n_1|\le 90 n_1 \log (43.3 n_1). \end{aligned}$$

Furthermore, using Lemma 18, we have

$$\begin{aligned} \max \left\{ Dh(b^{m_2-m_3}- 1),\left| \log (b^{m_2-m_3}- 1)\right| ,0.16\right\}&\le 2(m_2-m_3+1)\log b \\&\le 3.5 \log (43.3 n_1)\log b. \end{aligned}$$

Therefore we may choose $E=52 n_1$ in Theorem 16.

Now we obtain by Matveev’s theorem

$$\begin{aligned} 4.69\cdot 10^{9} \log (719 n_1)\log (43.3 n_1) (\log b)^2 \ge -\log |\Lambda | \end{aligned}$$

and then

$$\begin{aligned} 4.69\cdot 10^{9} (\log (719 n_1) \log b)^2 \ge -\log |\Lambda |. \end{aligned}$$

Together with the upper bound for $|\Lambda |$ this yields

$$\begin{aligned} 4.69\cdot 10^{9} (\log (719 n_1) \log b)^2 \ge \epsilon \log \alpha - \log (200n_1) \end{aligned}$$

and thus

26 $$\begin{aligned} \log \alpha < 4.7 \cdot 10^{9} \frac{1}{\epsilon }(\log (719 n_1) \log b)^2. \end{aligned}$$

Similar as in Sect. 7 we plug in this upper bound for $\log \alpha$ into (11) and obtain the inequality

$$\begin{aligned} 719 n_1< 2.17\cdot 10^{34} \epsilon ^{-2} (\log b \log (719 n_1))^4. \end{aligned}$$

Writing $n'=719 n_1$ and applying Lemma 22 gives us an upper bound for $n'$ and in the sequel for $n_1$, namely

$$\begin{aligned} n_1 < 4.83\cdot 10^{32} \frac{(\log b)^4}{\epsilon ^2} \left( \log \left( 5.56\cdot 10^{36} \frac{(\log b)^4}{\epsilon ^2}\right) \right) ^4. \end{aligned}$$

This concludes the proof of Theorem 1.

Now let us assume that assumption A2 holds. Then we put $\epsilon =\frac{1}{2}$ and get

$$\begin{aligned} n_1< 1.94\cdot 10^{33}(\log b)^4 \left( \log \left( 2.23\cdot 10^{37}(\log b)^4 \right) \right) ^4, \end{aligned}$$

in particular $n_1 < 2.3\cdot 10^{40}$ for $b=2$ (cf. Corollary 3). If we insert this upper bound into (26) with $\epsilon =\frac{1}{2}$, then we obtain

$$\begin{aligned} \log \alpha < 9.4\cdot 10^{9}\left( \log \left( 1.4\cdot 10^{36}(\log b)^4\left( \log \left( 2.23\cdot 10^{37}(\log b)^4\right) \right) ^4\right) \log b\right) ^2 \end{aligned}$$

which finally proves Theorem 2.

## Data Availability

This manuscript has no associated data.

## Footnotes

1 This prime *p* is called a *primitive divisor.*
